# Ambient fine particulate air pollution triggers ST-elevation myocardial infarction, but not non-ST elevation myocardial infarction: a case-crossover study

**DOI:** 10.1186/1743-8977-11-1

**Published:** 2014-01-02

**Authors:** Blake Gardner, Frederick Ling, Philip K Hopke, Mark W Frampton, Mark J Utell, Wojciech Zareba, Scott J Cameron, David Chalupa, Cathleen Kane, Suresh Kulandhaisamy, Michael C Topf, David Q Rich

**Affiliations:** 1 Division of Cardiology, Department of Medicine, University of Rochester Medical Center, Rochester, NY, USA; 2 Institute for a Sustainable Environment, Clarkson University, Potsdam, NY, USA; 3 Division of Pulmonary and Critical Care Medicine, Department of Medicine, University of Rochester Medical Center, Rochester, NY, USA; 4 Department of Public Health Sciences, University of Rochester School of Medicine and Dentistry, 265 Crittenden Boulevard, CU 420644, Rochester, NY, USA

**Keywords:** Myocardial infarction, Acute coronary syndrome, Epidemiology, Air pollution

## Abstract

**Background:**

We and others have shown that increases in particulate air pollutant (PM) concentrations in the previous hours and days have been associated with increased risks of myocardial infarction, but little is known about the relationships between air pollution and specific subsets of myocardial infarction, such as ST-elevation myocardial infarction (STEMI) and non ST-elevation myocardial infarction (NSTEMI).

**Methods:**

Using data from acute coronary syndrome patients with STEMI (n = 338) and NSTEMI (n = 339) and case-crossover methods, we estimated the risk of STEMI and NSTEMI associated with increased ambient fine particle (<2.5 um) concentrations, ultrafine particle (10-100 nm) number concentrations, and accumulation mode particle (100-500 nm) number concentrations in the previous few hours and days.

**Results:**

We found a significant 18% increase in the risk of STEMI associated with each 7.1 μg/m^3^ increase in PM_2.5_ concentration in the previous hour prior to acute coronary syndrome onset, with smaller, non-significantly increased risks associated with increased fine particle concentrations in the previous 3, 12, and 24 hours. We found no pattern with NSTEMI. Estimates of the risk of STEMI associated with interquartile range increases in ultrafine particle and accumulation mode particle number concentrations in the previous 1 to 96 hours were all greater than 1.0, but not statistically significant. Patients with pre-existing hypertension had a significantly greater risk of STEMI associated with increased fine particle concentration in the previous hour than patients without hypertension.

**Conclusions:**

Increased fine particle concentrations in the hour prior to acute coronary syndrome onset were associated with an increased risk of STEMI, but not NSTEMI. Patients with pre-existing hypertension and other cardiovascular disease appeared particularly susceptible. Further investigation into mechanisms by which PM can preferentially trigger STEMI over NSTEMI within this rapid time scale is needed.

## 

Previous studies investigating triggering of myocardial infarction by particulate air pollution (PM) concentrations have, in most cases, reported an increased risk of myocardial infarction associated with increases in PM on the same and previous day [1-9]. Similar acute effects of fine particulate air pollution have been reported for other cardiovascular outcomes [10,11]. Some studies of myocardial infarction and PM have used symptom onset time, rather than the arrival time at the emergency room, to define myocardial infarction onset, thereby providing a better estimate of the myocardial infarction onset time and less exposure error [1,4,5,7]. Although Peters et al. [4] reported a significantly increased risk of myocardial infarction associated with increased fine particle (particles <2.5 μm in diameter; PM_2.5_) concentrations in the preceding 2 hours, a second study by Peters et al. [5] as well as other studies exploring associations at these short lags, did not [1,5,7].

Recently, using hospital admissions data (without symptom onset time data), we reported that myocardial infarction/PM_2.5_ associations may be limited to transmural infarctions [6]. We found a 10% increased risk of transmural infarctions (95% CI = 1%, 20%), but not non-transmural infarctions (-1%; 95% CI = -6%, 5%), associated with each 10.8 μg/m^3^ increase in the PM_2.5_ concentration in the preceding 24 hours. We hypothesized that this may be due to differences in response to air pollution by myocardial infarction type. However, transmural and non-transmural myocardial infarction are estimations of assumed injury to the myocardium, whereas acute coronary syndromes attempt to describe the spectrum of physiologic events occurring in coronary arteries during an acute ischemic event. Therefore, in order to better understand the relationship between the acute pathophysiologic process of myocardial infarction and increased air pollutant concentrations, we sought to study acute coronary syndromes (i.e. ST segment elevation myocardial infarction [STEMI], non-ST segment elevation myocardial infarction [NSTEMI], and unstable angina) to reflect the spectrum of pathophysiologic events occurring. However, unstable angina was not included in this study since symptom onset times were not available, and the diagnosis is typically based on clinical judgment without objective criteria. STEMI and NSTEMI have clearly distinct objective data leading to their diagnosis, and symptom onset times were available.

STEMI is most often the result of plaque rupture followed by thrombus formation and coronary artery lumen occlusion. In STEMI, the thrombus is a terminal event with few arteries undergoing spontaneous recanalization. NSTEMI typically occurs as a consequence of excessive plaque burden and myocardial oxygen demand/supply mismatch, or plaque rupture and thrombus formation without complete coronary artery occlusion. At times, NSTEMI may include an occlusive thrombus or a transiently occlusive thrombus depending on available collateral vessels and myocardial metabolic demand [12]. Unstable angina is pathophysiologically similar to NSTEMI without the requirement of myocardial cell death during myocardial ischemia.

With plaque rupture, the balance between factors promoting thrombus formation and degradation is altered, which determines whether the vessel is completely or only partially occluded (i.e. STEMI vs. NSTEMI). Thus, a difference in acute coronary syndrome subtype (STEMI vs. NSTEMI) triggering by PM_2.5_ may suggest the mechanisms (e.g. coagulation, inflammation, etc.) most important for explaining how PM triggers myocardial infarction.

Using data (2007–2010) on acute coronary syndrome patients treated at the University of Rochester Medical Center (URMC) Cardiac Catheterization Laboratory, ambient air pollutant concentrations measured in Rochester, New York, and case-crossover methods, we addressed the hypothesis that increased PM_2.5_ (<2.5 μM in aerodynamic diameter) concentrations, and ultrafine particle (10-100 nm), and accumulation mode particle (100-500 nm) number concentrations in the previous 24 hours are associated with an increased risk of STEMI, but not NSTEMI. Using acute coronary syndrome patients’ self-reported symptom onset time, we also explored whether air pollutant concentrations at times more proximal to symptom onset of the acute coronary syndrome (e.g. 12 hours, 3 hours, and 1 hour before onset) would be associated with a larger increased risk. To do this, we used a case-crossover study [13,14], which is analogous to a matched case–control study. However, instead of contrasting air pollutant concentrations between people with disease (cases) and without disease (controls), we contrast pollutant concentrations from time periods right before an acute coronary syndrome event (case period) to time periods when that same person did not have an acute coronary syndrome event (control periods).

## Methods

### Study population and outcome definition

Since 2007, the URMC Cardiac Catheterization Laboratory has stored and maintained a database of all procedures and events treated, including acute coronary syndromes. This database includes information both on the subject (age, race, gender, residential address, previous co-morbidities and procedures, etc.) and the clinical event (i.e. onset date and time, acute coronary syndrome type, etc.).

The acute coronary syndromes in this dataset, seen in the URMC Cardiac Catheterization Laboratory, were defined using the current American College of Cardiology (ACC)/American Heart Association (AHA) guidelines [15]. For STEMI, on the presenting EKG, this is defined as ST segment elevation greater than 1 mm in 2 or more contiguous precordial leads, or 2 or more adjacent limb leads, or new or presumed new left bundle branch block in the appropriate clinical setting (angina or angina equivalent). For NSTEMI, the diagnosis is made by appearance of detectable cardiac myocyte biomarkers (indicating myocardial necrosis) in the blood of a patient without the requirement of distinctly dynamic changes on the EKG. For STEMI or STEMI equivalents (appearance of a new left bundle branch block), EKG criteria is both necessary and sufficient for the diagnosis in a patient deemed by the treating physician to have symptoms consistent with cardiac chest pain.

Onset time (date and hour) was self-reported by each patient (or kin if patient was unable to communicate) upon arrival to the URMC Cardiac Catheterization Laboratory. Only patients that presented to the URMC Cardiac Catheterization Laboratory were included in this study. However, unstable anginas were not included since the diagnosis is typically based on clinical judgment without objective criteria, and symptom onset times were not available.

From these acute coronary syndrome data, we retained all STEMI and NSTEMI events occurring from January 1, 2007 to December 31, 2010 (N = 3889), where symptom onset date and time were available, and those where the patient resided within 15 miles of our pollutant monitoring station in Rochester, New York, resulting in n = 338 STEMI and n = 339 NSTEMI available for analysis. This study was approved by the University of Rochester Medical Center Research Subjects Review Board.

### Air pollution and meteorology measurements

Pollutant concentration data used in the study were measured at the New York State Department of Environmental Protection site in Rochester, New York, located approximately 1500 m from an interstate highway. Particle number concentrations in the 10–500 nm diameter range were measured using a Scanning Mobility Particle Sizer (SMPS, TSI, Inc., Shoreview, MN), [16] which were used to generate particle number concentrations of ultrafine particles (≤100 nm) and accumulation mode particles (100-500 nm) for each hour during the study period (January 1, 2007 to December 31, 2010). PM_2.5_ was measured continuously using a Tapered Element Oscillating Microbalance (TEOM; ThermoFisher, Franklin, MA). Hourly temperature and relative humidity data were also measured at the site. These hourly pollutant concentration and weather data were used in all statistical analyses described below.

### Study design

We used a time-stratified case-crossover design [13,14], which we and others have used in studies of ambient air pollution and myocardial infarction [6-9]. This design is analogous to a matched case–control study. However, instead of contrasting air pollutant concentrations between a person experiencing a acute coronary syndrome (case) and a person who did not (control), it contrasts pollutant concentrations right before the acute coronary syndrome (case-period) to other periods when the subject did not have a acute coronary syndrome, matched to the case-period by calendar month, weekday, and hour of the day (control periods). Since the case and control periods are within the follow-up time of the same person, non-time varying confounders such as health history and smoking status are controlled by design. Factors that vary between case and control time periods (e.g. weather variables) are possible confounders that need to be included in our statistical models.

### Statistical analysis – main analysis

Using a conditional logistic regression model stratified on each acute coronary syndrome (one case and three or four control periods), we regressed STEMI case–control status (i.e., case period = 1, control period = 0) against the mean PM_2.5_ concentration in the 24 hours prior to symptom onset (lag hours 0–23). Using Akaike’s information criterion to select the optimal lag time and functional form (natural spline with 2, 3, or 4 degrees of freedom versus 1 degree of freedom/linear), we included the mean temperature and mean relative humidity in the previous 3 hours (1 degree of freedom). From this model, we present the risk of STEMI associated with each interquartile range increase in the mean PM_2.5_ concentration in the previous 24 hours and its 95% confidence interval. We also explored the risk of STEMI associated with each interquartile range increase in the mean PM_2.5_ concentration at shorter lag times (lag hours 0–11, 0–2, and 0) and longer lag times (0–47, 0–71, and 0–95), as well as with increased ultrafine particle and accumulation mode particle number concentrations at the same lag times, in the same manner. We then repeated these analyses for NSTEMI.

We also explored whether any increased risk of STEMI or NSTEMI associated with increased PM_2.5_ concentrations, ultrafine particle or accumulation mode particle number concentrations in the previous few hours or days was modified by a subject’s health status (i.e. pre-existing co-morbidities [e.g. hypertension, dyslipidemia, diabetes, low left ventricular ejection fraction], prior procedures including coronary artery bypass graft [CABG], percutaneous coronary intervention [PCI], myocardial infarction, peripheral artery disease), or subject characteristics (i.e. current vs. non-smoker; age, gender, race). To do this, we used the same conditional logistic regression model described above, adding an interaction term for the pollutant concentration and characteristic (e.g. PM_2.5_*SMOKER) to the model.

### Sensitivity analysis

Next, we evaluated whether restricting our study population to only those living within 5 miles of the air pollution monitoring site in Rochester would result in less exposure misclassification and therefore larger risk estimates associated with the same interquartile range increases in ambient PM_2.5_ concentration, ultrafine particle, and accumulation mode particle number concentrations. We ran the same conditional logistic regression models described above and compared the risk estimates from this model to that from the main analysis described above. We used SAS version 9.32 (©SAS Institute, Inc. Cary, NC) to construct all datasets and conduct descriptive analyses, and R (version 2.15.1; R Foundation for Statistical Computing, Vienna, Austria) to perform all conditional logistic regression analyses.

## Results

There was little difference between STEMI and NSTEMI patients by gender (66% and 68% male respectively), age (STEMI: mean ± standard deviation = 61 ± 12 years; NSTEMI: mean ± standard deviation = 63 ± 14 years), and ethnicity (STEMI: 85% white, NSTEMI: 85% white; Table 1). Similarly, 41% of STEMI and 39% of NSTEMI patients had left ventricular ejection fractions < 45%. NSTEMI patients were, however, more likely to have had a prior myocardial infarction, coronary artery bypass graft surgery, a cerebrovascular event, more likely to have previously been diagnosed with dyslipidemia, diabetes, heart failure, and chronic lung disease, and more likely to be obese or severely obese than STEMI patients. However, STEMI patients were more likely to have been smokers at the time of their myocardial infarction (65%) compared to NSTEMI patients (31%).

**Table 1:** Patient characteristics

**Characteristic**	**STEMI**		**NSTEMI**	
	**N = 338**	**%**	**N = 339**	**%**
**Age**				
<50 years	58	17%	58	17%
50-59 years	106	31%	94	28%
60-69 years	92	27%	80	24%
70-79 years	46	14%	54	16%
≥ 80 years	34	10%	53	15%
Missing age	2	1%	0	0%
**Male**	224	66%	229	68%
**Race**				
White	207*	87%	240**	86%
Black	27	11%	33	12%
Other	4	2%	7	3%
**Clinical presentation (may have more than 1)**				
**Study subjects with data on ACS presentation**	**241**	71%*	**280**	83%**
Prior myocardial infarction	54	22%	75	27%
Prior PCI	45	19%	56	20%
Prior CABG	10	4%	33	12%
CVD (“Stroke” in 07–08, “CVD” in 09–10)	12	5%	31	11%
Prior PAD	16	7%	25	9%
Smoking	156	65%	87	31%
Hypertension	160	66%	176	63%
Dyslipidemia	143	59%	199	71%
Diabetes	44	18%	65	23%
Prior HF	6	2%	26	9%
**BMI**				
Overweight (25 kg/m^2^ < = BMI <30 kg/m^2^)	99	41%	86	31%
Obesity ( BMI ≥30 kg/m^2^)	52	22%	80	29%
Severe obesity (BMI ≥35 kg/m^2^)	28	12%	42	15%
Mean ± STD	28 ± 5		30 ± 12	
**Left ventricular ejection fraction**				
≤35%	48	20%	54	19%
≤45%	98	41%	110	39%
Mean ± STD	43 ± 14		45 ± 14	

*STEMI: Total n = 238 with race/ethnicity information. There were n = 100 (30% of n = 338) with missing race/ethnicity.

**NSTEMI: Total n = 280 with race/ethnicity information. There were n = 59 (17% of n = 339) with missing race/ethnicity.

**Figure 1:**
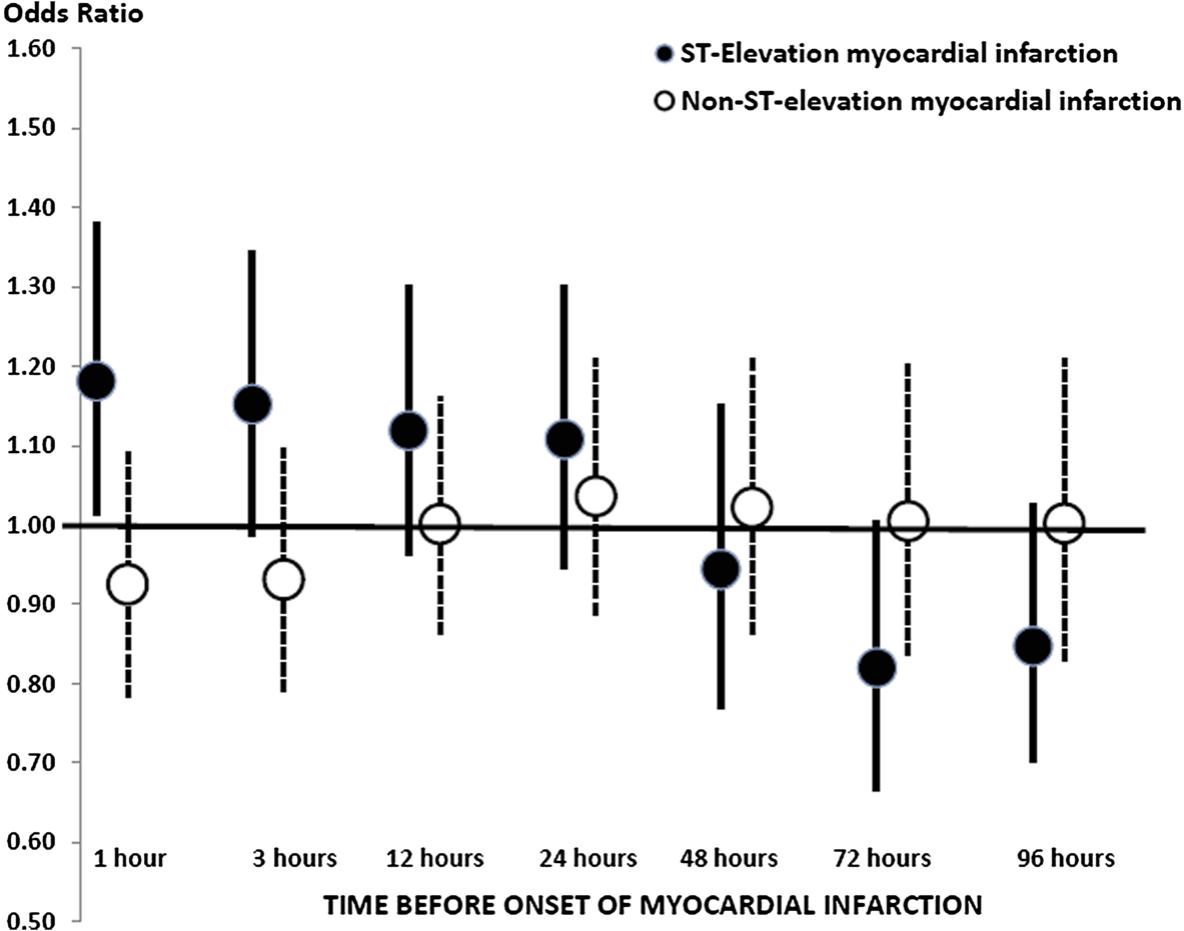
**Risk (and 95% confidence interval) of STEMI and NSTEMI associated with each interquartile range increase in PM**_**2.5**_**concentration, by lag hours.**

The distributions of ambient fine particle (PM_2.5_) concentrations, and ultrafine and accumulation mode particle number concentrations, as well as temperature and relative humidity in Rochester, NY during the study period (January 2007 to December 2010) are shown in Table 2. Included in Table 2 are the interquartile ranges of each pollutant’s hourly concentrations, which we used to scale the 1 hour risk estimates in Tables 3 and 4 and Figure 1. Also presented are the interquartile ranges of the absolute differences between case and control period pollutant concentrations, by which Kunzli and Schindler previously suggested scaling odds ratio estimates in case-crossover studies [17].

**Table 2:** Distribution of hourly pollutant concentrations and weather characteristics during the study period (January 1, 2007 to December 31, 2010)

**Pollutant/weather parameter**	**n**	**Mean**	**Standard deviation**	**Minimum**	**25**^**th**^	**50**^**th**^	**75**^**th**^	**Maximum**	**IQR**	**Case–control IQR**
PM_2.5_	1283	8.00	5.70	0.27	3.9	6.7	10.2	43.0	6.3	5.3
(μg/m^3^)										
Ultrafine particles	1231	5466	2,865	480	3503	5029	6874	37,291	3371	3116
(#/cm^3^)										
Accumulation mode particles	1231	996	643	108	501	849	1322	4444	821	713
(#/cm^3^)										
Temperature	1460	10	10	-13	2	11	20	30	18	5
(°C)										
Relative humidity	1461	65	12	30	57	66	74	94	17	11
(%)										

N = 1461 possible days.

**Table 3:** Risk (and 95% confidence intervals) of STEMI and NSTEMI associated with each interquartile range increase in pollutant concentration

			**STEMI**				**NSTEMI**			
**Pollutant**	**Lag hours**	**IQR**	**n**	**Odds ratio**	**95% confidence interval**	**p-value**	**n**	**Odds ratio**	**95% confidence interval**	**p-value**
	0	7.1	338	1.18	1.01-1.38	0.04	339	0.93	0.78-1.09	0.36
	0-2	6.8	335	1.15	0.99-1.35	0.07	338	0.93	0.79-1.10	0.39
PM_2.5_	0-11	6.1	333	1.12	0.95-1.31	0.17	338	1.00	0.85-1.17	0.99
(μg/m^3^)	0-23	5.9	329	1.11	0.93-1.32	0.24	335	1.04	0.88-1.22	0.68
	0-47	5.4	332	0.94	0.79-1.13	0.53	330	1.02	0.85-1.22	0.81
	0-71	5.1	318	0.82	0.67-1.00	0.05	327	1.00	0.83-1.21	0.96
	0-95	5.1	314	0.85	0.69-1.05	0.12	328	1.00	0.82-1.23	0.99
	0	4245	270	1.03	0.95-1.12	0.51	283	0.99	0.90-1.10	0.88
	0-2	4159	275	1.00	0.89-1.13	0.99	284	1.02	0.92-1.13	0.69
UFP	0-11	3475	274	1.05	0.90-1.23	0.53	284	1.04	0.91-1.20	0.60
(particles/cm^3^)	0-23	3284	274	1.06	0.89-1.26	0.50	276	1.01	0.86-1.18	0.90
	0-47	2989	273	1.01	0.84-1.21	0.92	277	0.96	0.80-1.16	0.70
	0-71	2834	274	1.03	0.84-1.25	0.80	279	0.93	0.75-1.15	0.49
	0-95	2742	278	1.06	0.86-1.30	0.60	278	0.89	0.71-1.12	0.33
	0	860	270	1.07	0.91- 1.27	0.39	283	0.97	0.82-1.15	0.70
	0-2	871	275	1.03	0.86-1.23	0.75	284	0.96	0.80-1.14	0.62
AMP	0-11	840	274	1.11	0.91-1.36	0.32	284	0.97	0.80-1.17	0.73
(particles/cm^3^)	0-23	775	274	1.12	0.92- 1.38	0.26	284	0.97	0.81-1.17	0.78
	0-47	673	273	1.10	0.90- 1.35	0.33	277	0.94	0.78-1.12	0.48
	0-71	615	274	1.04	0.85- 1.27	0.69	279	0.92	0.76-1.10	0.34
	0-95	558	278	1.04	0.86- 1.26	0.67	278	0.89	0.74-1.07	0.21

**Table 4:** **Risk (and 95% confidence intervals) of STEMI associated with each 7.1 μg/m**^**3**^**increase in PM**_**2.5**_**concentration in the previous hour**

**Characteristic**	**Category**	n*	**Odds ratio**	**95% confidence interval**	**p-value**	**Interaction term p-value**
Gender	Male	224	1.11	0.91-1.35	0.30	0.27
	Female	114	1.31	1.03-1.65	0.03	
Age	< 65 years	220	1.09	0.90-1.32	0.36	0.14
	≥ 65 years	118	1.37	1.07-1.75	0.01	
Race	White	207	1.15	0.94-1.41	0.18	0.83
	Other	32	1.21	0.78-1.89	0.40	
Smoker	Yes	104	1.07	0.81-1.41	0.65	0.75
	No	106	1.22	0.92-1.60	0.17	
Prior myocardial infarction	Yes	54	1.52	1.04-2.22	0.03	0.11
	No	185	1.08	0.87-1.33	0.49	
Prior CABG	Yes	10	1.27	0.60-2.66	0.53	0.80
	No	229	1.15	0.95-1.40	0.15	
Prior PCI	Yes	45	1.37	0.91-2.05	0.13	0.36
	No	195	1.11	0.90-1.37	0.33	
Prior PAD	Yes	16	1.31	0.66-2.61	0.45	0.76
	No	222	1.17	0.96-1.43	0.12	
Hypertension	Yes	160	1.39	1.10-1.76	<0.01	0.02
	No	76	0.90	0.66-1.23	0.51	
Left ventricle ejection fraction	< 35%	48	1.35	0.94-1.93	0.10	0.37
	≥ 35%	133	1.11	0.85-1.43	0.44	
Diabetes	Yes	44	1.15	0.75-1.76	0.53	0.98
	No	152	1.16	0.91-1.46	0.23	
Dyslipidemia	Yes	143	1.24	0.97-1.58	0.09	0.60
	No	94	1.12	0.85-1.49	0.42	

*Of the total possible N = 338 STEMI patients, some had missing data on subject characteristics and thus were not included in the specific stratified analysis.

Hourly PM_2.5_ concentrations were moderately correlated with accumulation mode particle number concentrations (r = 0.59), but were not correlated with ultrafine particle number concentrations (r = 0.09), temperature (r = 0.08), or relative humidity (r = 0.05). Hourly ultrafine particle number concentrations were weakly correlated with accumulation mode particle number concentrations (r = 0.32), and not correlated with temperature (r = 0.02) or relative humidity (r = -0.17). Similarly, hourly accumulation mode particle number concentrations were not correlated with temperature (r = 0.19) or relative humidity levels (r = -0.03) during the study period.

Next we estimated the risk of STEMI and NSTEMI associated with each interquartile range increase in PM_2.5_, ultrafine, and accumulation mode particle concentrations in the previous 1, 3, 12, 24, 48, 72, and 95 hours (Table 3; Figure 1). We found a significant 18% increase in the risk of STEMI associated with each 7.1 μg/m^3^ increase in PM_2.5_ concentration in the previous hour, with smaller, non-significant increases in the risk of STEMI associated with each interquartile range increase in PM_2.5_ concentration in the previous 3 hours (15%), 12 hours (12%), and 24 hours (11%). Estimates of the risk of STEMI associated with interquartile range increase increases in PM_2.5_ concentrations in the previous 48, 72, and 96 hours were all less than 1.0. We found no such pattern with NSTEMI with all risk estimates close to 1.0 for all PM_2.5_ concentration averaging times before myocardial infarction onset. Estimates of the risk of STEMI associated with interquartile range increases in ultrafine particle and accumulation mode particle number concentrations in the previous 1 to 96 hours were all greater than 1.0, but none were statistically significant.

The risks of STEMI associated with each 7.1 μg/m^3^ increase in PM_2.5_ concentration in the previous hour, stratified by patient characteristics, are shown in Table 4. STEMI patients with hypertension had a significantly larger risk of STEMI associated with each 7.1 μg/m^3^ increase in PM_2.5_ concentration in the previous hour than those without hypertension. Although not significantly different, patients with dyslipidemia, left ventricular ejection fractions <35%, prior peripheral arterial disease, prior percutaneous coronary intervention, prior coronary artery bypass graft, prior myocardial infarction, and patients who were non-smokers at the time of the myocardial infarction, not white, ≥ 65 years of age, and female had larger risks of STEMI associated with increased PM_2.5_ concentration in the previous hour than those without these conditions or those who were white, < 65 years of age, or male.

Last, we evaluated whether including only those STEMI patients residing within 5 miles of the pollutant monitoring station would result in less exposure error, less bias, and therefore larger risk of STEMI estimates than in our main analysis (i.e. residence within 15 miles of monitoring site). When including only those STEMI patients living within 5 miles of the pollutant monitoring station, the risk of STEMI associated with IQR increases in PM_2.5_ in the previous hour (OR = 1.17; 95% CI = 0.98, 1.40) and previous 24 hours (OR = 1.12; 95% CI = 0.92, 1.36) were similar to those from the main analysis including myocardial infarction patients within 15 miles of the site (1 hour OR = 1.18, 95% CI = 1.01, 1.38; 24 hour OR = 1.11, 95% CI = 0.93, 1.32). Similarly, the risk estimates associated with interquartile range increase increases in ultrafine particle number concentrations in the previous 1 hour (OR = 1.01; 95% = 0.92, 1.11) and 24 hours (OR = 1.09; 95% CI = 0.89, 1.33), and with interquartile range increase increases in accumulation mode particle number concentrations in the previous 1 hour (OR = 1.15; 95% = 0.96, 1.38) and 24 hours (OR = 1.17; 95% CI = 0.93, 1.46) were also similar to those from the main analysis.

## Discussion

Using data from the URMC Cardiac Catheterization Laboratory with information on the time of symptom onset, acute coronary syndrome subtype (STEMI or NSTEMI), and demographic and clinical variables, we found that increased ambient PM_2.5_ concentrations were associated with immediate (within 1 hour) increases in the risk of STEMI, but not NSTEMI. Effect estimates were largest for those with prior cardiovascular disease/events, specifically those with pre-existing hypertension, and non-smokers, those 65 years and older, Caucasians, and women. This finding of fine particle triggering of STEMI, but not NSTEMI, within 1 hour, suggests potential mechanisms by which this response could occur, and also that these mechanisms must act on this rapid time scale. Such mechanisms might result in more extensive plaque rupture or the promotion of thrombus formation in the subsequent interplay between prothrombotic and antithrombotic vascular processes.

Previous studies have reported an increased risk of myocardial infarction associated with increased PM_2.5_ and other pollutants in the preceding day or two [3]. Only our previous study [6] investigated PM associations by type of myocardial infarction, finding that increased PM_2.5_ concentration in the 24 hours before emergency room arrival for the myocardial infarction was associated with an increased risk of those infarctions progressing to a transmural infarction but not those progressing only as far as a partial wall infarction [6]. Only a few studies had symptom onset time data and thus were able to examine whether increased pollutant concentrations in the previous few hours, and not just the same day or just within 24 hours, triggered the myocardial infarction [1,4,5,7]. Only our current study and that of Peters et al. [4] report such an immediate response, while other studies do not [1,5,7]. This inconsistency may be due, at least in part, to the distribution of STEMI/NSTEMI within the sample of acute coronary syndrome included in each study. Our findings of greater risk of STEMI associated with increased PM_2.5_ concentrations among those subjects with pre-existing cardiovascular disease and/or prior acute cardiovascular events are consistent with findings from previous studies, [6,8,9] and affirm a hypothesis of increased susceptibility for those with pre-existing coronary artery disease.

There are multiple mechanisms for acute coronary syndrome and specifically STEMI and NSTEMI, including coagulation, inflammation, vascular dysfunction, and autonomic dysfunction. However, when a thrombus forms, circulating platelets are tethered via intermediate filaments to one another and to the injured vascular wall. Mechanistically, STEMI differ from NSTEMI in that STEMIs progress acutely to complete arterial occlusion following plaque rupture, whereas plaque-rupture mediated NSTEMIs do not. It is important to note that plaque rupture itself is necessary, but in isolation is insufficient to promote full arterial occlusion, and a cascade of coordinated cellular events must occur prior to thrombus formation. Furthermore, endogenous thrombolysis is an important protective process which can abrogate complete arterial occlusion (i.e. STEMI). Due to fundamental mechanistic differences between STEMI and NSTEMI, morbidity and mortality are higher with STEMI [18,19]. Thus, one may propose that the intracellular signal transduction mechanisms committing a patient to a STEMI distinguish it from NSTEMI by a different balance between thrombosis and thrombolysis. PM may be more likely to cause a STEMI than a NSTEMI if PM enhances the rate or extent of thrombus formation following plaque rupture and/or if PM exposure impairs thrombolysis, increasing the chance for complete vessel occlusion.

Plaque rupture and subsequent acute thrombosis of previously narrowed or unstable plaque-lined arteries is recognized as the proximal event in the evolution of myocardial infarction [20]. Previously, we reported large changes in circulating markers of platelet and endothelial cell activation (p-selectin, CD40L, von Willebrand factor [vWF]), in healthy young medical residents, associated with increased PM_2.5_ and other pollutants during the 2008 Beijing Summer Olympics [21,22]. We have also found increased platelet activation following controlled ultrafine particle exposure in diabetics [23]. We and others have found increased fibrinogen and C-reactive protein levels (inflammatory markers) associated with increased ambient PM_2.5_ concentrations, and accumulation mode and ultrafine particle number concentrations in the preceding few days, [22] especially in subjects with underlying cardiovascular risk factors [24,25]. However, some controlled human exposure studies have not reproduced these data [26,27]. Furthermore, changes in vWF, p-selectin, CD40L, and platelet aggregation have also been repeatedly associated with acute increases in PM concentration in both humans and animals, [25,27-30] suggesting these interacting coagulation/inflammation pathways may be acutely affected by PM air pollution exposure. Consistent with this, controlled exposure studies have reported impaired vascular function and increased thrombogenesis following diesel exhaust exposure [31-35]. Only a few studies have contrasted the circulating concentration of inflammatory and thrombotic biomarker levels in STEMI and NSTEMI from blood samples drawn upon arrival in the emergency room, and reported increases in thrombotic/inflammatory markers (e.g. WBC, C-reactive protein, ferritin, serum amyloid A) [36,37]. Other studies found no difference in these and other platelet markers (e.g. mean platelet volume, platelet count) [38,39]. However, of the biochemical and physiological mechanisms associated with ambient PM exposure, only thrombus formation and vasoconstriction occur on such a rapid time scale. Vascular constriction is an instantaneous response to offending stimuli mediated by endothelial and non-endothelial factors, whereas thrombus formation is a continuum of events that is initiated by platelet activation, aggregation, and adhesion to an injured vessel wall. This time continuum makes thrombus formation more variable, and may explain fundamental differences between STEMI and NSTEMI with respect to PM exposure.

Although our study had several strengths including well characterized acute coronary syndrome events in the Cardiac Catheterization Laboratory, there are several important limitations that should be considered when making inference. First, we had limited sample size due to our use of existing data, the requirement of symptom onset time data, and a residence within 15 miles of the monitoring site. This limited sample size resulted in reduced statistical power and limited precision in our risk estimates. Second, we used ambient PM_2.5_ concentrations, and ultrafine particle and accumulation mode particle number concentrations, measured at one central site monitoring location, to represent each myocardial infarction patient’s exposure to PM of outdoor origin, regardless of how close subjects lived, worked, or spent time to the monitoring site. This exposure error, however, is not likely different for time periods immediately preceding the myocardial infarction and other time periods in the weeks before or after the STEMI/NSTEMI, likely resulting in biases toward the null and underestimates of the risk. Further, when we included only those STEMI patients living within 5 miles of the monitoring site, our estimates of the risk of STEMI associated with increased PM_2.5_ concentration, ultrafine particle number concentration and accumulation mode particle number concentration in the previous hour were similar. Since UFP and AMP number concentrations are more spatially variable across space than PM_2.5_ concentrations, in part due to differences in sources (AMP formed by atmospheric chemistry oxidizing precursor gases like SO_2_ and NO_2_; UFP locally emitted and having a shorter atmospheric lifetime), it is not surprising they are not well correlated with PM_2.5_ concentrations [40]. Thus it is also not surprising we did not find increased risks of STEMI associated with increased UFP and AMP number concentrations.

Third, symptom onset time was determined by patient self-report upon arrival to the Cardiac Catheterization Laboratory. This is a reasonable surrogate marker for ischemia onset, but cannot be extrapolated to infarction onset with confidence. The temporal relationship between plaque rupture, ischemia and infarction may differ between individuals as much as it differs between STEMI and NSTEMI. If STEMI and NSTEMI have the same true OR associated with increased PM_2.5_ concentration (e.g. 1.50), but there is a greater degree of error in estimating NSTEMI onset time than STEMI onset time, the risk of NSTEMI associated with increased PM_2.5_ concentration could be underestimated to a greater degree (i.e. OR = 1.00) than the risk of STEMI associated with increased PM_2.5_ concentration (i.e. OR = 1.18). Further work is therefore needed to confirm that our findings were not due to this exposure error.

It is also possible that our finding is due to residual confounding by an unmeasured confounder. However, for such a factor to exist, it would have to vary temporally between weeks within each subject, be correlated with ambient air pollution levels, and also be a risk factor for myocardial infarction independent of air pollution. Outside of temperature and relative humidity, numerous studies examining the acute risk of cardiovascular events including myocardial infarction associated with air pollution exposure have not identified such a factor.

As shown in Table 1, STEMI patients were slightly younger, less likely to be obese, more likely to be smokers, and were less likely to have several cardiovascular co-morbidities or procedures than NSTEMI patients. Although not significantly different for most factors, STEMI patients with many of these factors had an even larger relative risk of STEMI associated with increased PM_2.5_ in the previous hour than STEMI patient without them. If these factors put one at increased risk for an ACS, then we would have expected to see an increased risk of NSTEMI associated with increased PM_2.5_ concentrations, rather than STEMI. Thus, although it is possible that our findings of PM_2.5_ triggering of STEMI but non NSTEMI might simply reflect differences in the population presenting rather than a different biological mechanism, our effect modification findings do not support this.

We only studied patients receiving care in the Cardiac Catheterization Laboratory of a tertiary care hospital. Thus our subjects may not be representative of the broader population of acute coronary syndrome patients that do not have access to cardiac catheterization, or who are medically managed. However, if our findings can be confirmed in a prospective study addressing many of the weaknesses described above, the findings can be generalized to all US adults who would be treated at a hospital’s cardiac catheterization laboratory, since plaque rupture with thrombus formation generally is the most prevalent cause of STEMI and NSTEMI [41].

## Conclusions

Using data from acute coronary syndrome patients with STEMI and NSTEMI, ambient measurements of particulate air pollution concentrations, and case-crossover methods, we found that increased PM_2.5_ concentrations in the hour prior to acute coronary syndrome onset were associated with an increased risk of STEMI, but not NSTEMI. Further investigation into mechanisms by which PM_2.5_ can preferentially trigger STEMI over NSTEMI within this rapid time scale is needed. These investigations may provide information needed to develop interventions to prevent air pollution mediated acute coronary syndromes.

## Abbreviations

ACC: American College of Cardiology; ACS: Acute coronary syndrome; AHA: American Heart Association; AMP: Accumulation mode particles; CABG: Coronary artery bypass graft; CI: Confidence interval; EKG: Electrocardiogram; NSTEMI: Non-ST-elevation myocardial infarction; OR: Odds ratio; PAD: Peripheral arterial disease; PCI: Percutaneous coronary intervention; PM2.5: Fine particles, particulate matter <2.5 μm in diameter; STEMI: ST-elevation myocardial infarction; TEOM: Tapered element oscillating microbalance; UA: Unstable angina; URMC: University of Rochester Medical Center.

## Competing interests

The authors have no competing interests.

## Authors’ contributions

BG, FL, MF, MU, WZ, and DR conceived and designed the study. PK and DC collected the air pollution data. BG, SK, and MT collected the acute coronary syndrome data from medical records. DR, BG, SC, MF, MU, FL, WZ, PK, and CK conducted and interpreted the statistical analyses. DR, BG, and SC drafted the manuscript. All authors read and approved the final manuscript.
